# Contribution of *CACNA1H* Variants in Autism Spectrum Disorder Susceptibility

**DOI:** 10.3389/fpsyt.2022.858238

**Published:** 2022-03-08

**Authors:** Marta Viggiano, Tiziano D'Andrea, Cinzia Cameli, Annio Posar, Paola Visconti, Maria Cristina Scaduto, Roberta Colucci, Magali J. Rochat, Fabiola Ceroni, Giorgio Milazzo, Sergio Fucile, Elena Maestrini, Elena Bacchelli

**Affiliations:** ^1^Department of Pharmacy and Biotechnology, University of Bologna, Bologna, Italy; ^2^Department of Physiology and Pharmacology, Sapienza University, Rome, Italy; ^3^Unità Operativa Semplice d'Istituto (UOSI) Disturbi dello Spettro Autistico, Istituto di Ricovero e Cura a Carattere Scientifico (IRCCS) Istituto delle Scienze Neurologiche di Bologna, Bologna, Italy; ^4^Department of Biomedical and Neuromotor Sciences, University of Bologna, Bologna, Italy; ^5^Department of Medical and Surgical Sciences, University of Bologna, Bologna, Italy; ^6^Functional and Molecular Neuroimaging Unit, Istituto di Ricovero e Cura a Carattere Scientifico (IRCCS) Istituto delle Scienze Neurologiche di Bologna, Bologna, Italy; ^7^Istituto di Ricovero e Cura a Carattere Scientifico (IRCCS) Neuromed, Pozzilli, Italy

**Keywords:** ASD, rare variants, VGCCs, *CACNA1H*, Ca_v_3.2, calcium channel

## Abstract

Autism Spectrum Disorder (ASD) is a highly heterogeneous neuropsychiatric disorder with a strong genetic component. The genetic architecture is complex, consisting of a combination of common low-risk and more penetrant rare variants. Voltage-gated calcium channels (VGCCs or Ca_v_) genes have been implicated as high-confidence susceptibility genes for ASD, in accordance with the relevant role of calcium signaling in neuronal function. In order to further investigate the involvement of VGCCs rare variants in ASD susceptibility, we performed whole genome sequencing analysis in a cohort of 105 families, composed of 124 ASD individuals, 210 parents and 58 unaffected siblings. We identified 53 rare inherited damaging variants in Ca_v_ genes, including genes coding for the principal subunit and genes coding for the auxiliary subunits, in 40 ASD families. Interestingly, biallelic rare damaging missense variants were detected in the *CACNA1H* gene, coding for the T-type Ca_v_3.2 channel, in ASD probands from two different families. Thus, to clarify the role of these *CACNA1H* variants on calcium channel activity we performed electrophysiological analysis using whole-cell patch clamp technology. Three out of four tested variants were shown to mildly affect Ca_v_3.2 channel current density and activation properties, possibly leading to a dysregulation of intracellular Ca^2+^ ions homeostasis, thus altering calcium-dependent neuronal processes and contributing to ASD etiology in these families. Our results provide further support for the role of *CACNA1H* in neurodevelopmental disorders and suggest that rare *CACNA1H* variants may be involved in ASD development, providing a high-risk genetic background.

## Introduction

Autism Spectrum Disorder (ASD) is a group of clinically heterogeneous neurodevelopmental disorders with a prevalence of >1% (1), characterized by impairments in communication and social interaction, and the presence of repetitive and restrictive behaviors (2).

ASD is a multifactorial disorder, with a strong genetic component and an estimated heritability of 60–90% (3, 4). The genetic architecture is highly heterogeneous and consists of a complex interplay of rare deleterious variants and common low-risk alleles. The discovery of rare, highly penetrant, variants in a proportion of cases (10–25%) (5), contributed to the identification of numerous candidate genes, showing functional convergence on a small set of common pathological pathways. Among these, calcium signaling has been consistently implicated in the molecular bases of ASD and associated comorbidities (6–8). It represents a universal and versatile pathway involved in a wide range of cellular processes including synaptic plasticity, by modulating neurotransmitter release and shaping of the synaptic membrane composition (9). Intracellular calcium concentration is mainly but not exclusively regulated by voltage-gated calcium channels (VGCCs or Ca_v_ channels), a family of calcium channels that allow the influx of ions into the cell in response to voltage changes, regulating intracellular calcium concentration and initiating a variety of calcium-dependent processes, such as exocytosis and neurotransmitter release (10). VGCCs variants have been indicated as a shared risk factor for several neuropsychiatric disorders (11). In particular, perturbation of intracellular calcium homeostasis caused by disruption of VGCCs genes has been associated to increased ASD susceptibility (8, 9, 12).

In order to investigate the contribution of VGCCs to ASD, we analyzed the sequence of the whole genome in a cohort of 105 families comprising 124 individuals with a diagnosis of ASD, and we looked for rare coding damaging variants in genes encoding for VGCCs. We evaluated variants in genes for the VGCCs α1 principal subunit (*CACNA1A, CACNA1B, CACNA1C, CACNA1D, CACNA1E, CACNA1F, CACNA1G, CACNA1H, CACNA1I, CACNA1S*), as well as genes coding for the auxiliary subunits (*CACNA2D1, CACNA2D2, CACNA2D3, CACNA2D4, CACNB1, CACNB2, CACNB3* and *CACNB4*). Auxiliary subunits have also been implicated in ASD risk, due to their important role in the regulation of channel biophysical properties and targeting of the α1 subunit to the cell membrane (13).

Interestingly, we identified biallelic variants in the *CACNA1H* gene in two unrelated ASD families. *CACNA1H* encodes the Ca_v_3.2 channel, belonging to the low-voltage-activated (LVA) T-type Ca_v_ channels subfamily, that is widely expressed in mammalian tissues, including brain where is involved in the regulation of neuronal firing (14). *CACNA1H* missense variants have been previously identified in ASD individuals and implicated in the ASD phenotype (15). Thus, we used heterologous expression of mutated Ca_v_3.2 channels in mammalian cells to perform electrophysiological analysis of the identified *CACNA1H* variants, to functionally characterize their impact and to assess if the ASD phenotype could be explained by the combined effect of the two mutations in the gene.

## Materials and Methods

### Cohort

Our cohort consists of 105 families with ASD, recruited at the UOSI Disturbi dello Spettro Autistico, IRCCS Istituto delle Scienze Neurologiche (Bologna, Italy).

The sample includes 21 multiplex and 84 simplex families, for a total of 98 males and 26 females with ASD, as well as 210 parents and 58 unaffected siblings. DNA samples were extracted from whole blood.

Individuals with ASD were assessed using a set of standardized diagnostic instruments to evaluate the ASD phenotype (ADOS, CARS and M-CHAT), to assess developmental/cognitive levels (PEP-3, Leiter-R, Griffith Scales, or Wechsler Scales) and adaptive behavior (Vineland Adaptive Behavior Scale, VABS); clinical signs such as mimicry, hyperactivity, sensory abnormalities and symptoms onset were also evaluated. Moreover, probands underwent EEG and MRI. Subclinical features in relatives were assessed using the Social and Communication Disorders Checklist and The Broad Autism Phenotype Questionnaire.

All participants provided a written informed consent to participate to this study. This study was approved by the local Ethical Committee (Comitato Etico di Area Vasta Emilia Centro (CE-AVEC); code CE 14060). All research was performed in accordance with the relevant guidelines and regulations.

### Whole Genome Sequencing Analysis

Whole genome sequencing (WGS) was performed at New York Genome Center. Quality controls, alignment and variant calling were carried out according to the pipeline developed by the Center for Common Disease Genomics project (https://github.com/CCDG/Pipeline-Standardization/blob/master/PipelineStandard.md).

Variant annotation was performed with ANNOVAR, using RefSeq for gene-based annotation (Genome Build hg38). Annotated variants were filtered in order to retain only coding and splicing variants, and to remove low-quality variants [Coverage (DP) <10 and Genome Quality (GQ) <20]. To select rare variants, a minor allele frequency (MAF) threshold ≤ 1% in Genome Aggregation Database (gnomAD, https://gnomad.broadinstitute.org/) was chosen. Specifically, population allele frequencies for variants were obtained from the non-neurological subset of gnomAD v.2.1 and the entire data set of gnomAD v.3.0, which represents a large collection of individuals of different ancestry. Variants were further filtered according to their exonic function, excluding synonymous variants and highlighting deleterious variants, including Likely Gene Disrupting (LGD) and damaging missense variants. LGD variants consist of stop-gain, stop-loss, frameshift and splicing variants, while damaging missense variants were defined according to CADD score (16), assuming CADD score value ≥ 15 as damaging threshold.

To identify variants acting under a recessive inheritance model, homozygous, hemizygous and compound heterozygous variants in probands were selected.

Ultra-rare variants were obtained by further filtering variants according to their MAF in the same previously used data sets, but retaining only variants having MAF ≤ 0.1%.

Genes previously associated with ASD were defined using the SFARI Gene database and its scoring system, including four categories: S (syndromic), 1 (high confidence), 2 (strong candidate) and 3 (suggestive evidence) (https://gene.sfari.org/, Release: 2021 Q3).

### Functional Analysis of ASD Variants on Ca_v_3.2 Protein Activity

#### Generation of WT and Mutant Plasmid Constructs

Recombinant WT protein expression construct for CACNA1H was generated by cloning the coding sequence (NM_021098.3), in frame with the 3xFLAG epitope, in the p3xFLAG-CMV-10 mammalian expression vector (Sigma-Aldrich). CACNA1H coding sequence was subcloned into p3xFLAG-CMV-10 from a1Ha-pcDNA3 plasmid (Addgene plasmid #45809; http://n2t.net/addgene:45809; RRID:Addgene_45809) (17). WGS identified mutations [p.(Lys785Met), p.(Pro849Ser), p.(Pro2124Leu), p.(Ser2338Phe)] were introduced to the WT CACNA1H coding sequence by multi-step site-directed mutagenesis, performing whole-plasmid PCR reactions (primers are reported in Supplementary Materials) with Herculase II Fusion DNA Polymerase (Agilent Technologies, Inc.). Sanger sequencing was performed to check both WT and mutant coding sequences cloned (BigDye Terminator Cycle Sequencing kit-ThermoFisher Scientific).

#### Transient Transfection and Immunofluorescence (IF) Assay

Human Embryonic Kidney-293T (HEK-293T) cells were grown on collagen coated glass coverslips in 6-well plate in Dulbecco's Modified Eagle's Medium (DMEM) high glucose (Sigma-Aldrich) supplemented with 10% fetal bovine serum and 0.05 mg/ml penicillin-streptomycin (DMEM-complete medium), at 37°C in a 5% CO_2_ humidified atmosphere.

Cells were transiently transfected with 3 μg of p3xFLAG-CACNA1H plasmid constructs using 0.3 μl of PolyEthylenImine (PEI) *per* 1 μg of plasmid DNA in DMEM + L-glutamine (1mM). Ninety minutes after transfection, serum deprived medium was replaced with DMEM-complete medium and cells were maintained at 37°C in a 5% CO_2_ humidified atmosphere. GFP-coding plasmid was used as positive transfection control, while a p3xFLAG-CMV-10 empty vector and a 3xFLAG plasmid encoding the 3xFLAG-ABCC3 transmembrane fusion protein were used as IF negative and positive control respectively.

Forty-eight hours after transfection, cells were fixed for 15' with 4% paraformaldehyde in PBS, washed three times in PBS and incubated with blocking solution (4% normal donkey serum and 0.05% tween in PBS) for 30' at room temperature (RT). Then, cells were stained with mouse anti-FLAG M2 antibody (1:200; Sigma-Aldrich) for 1.5 h at RT. After three 0.05% tween-PBS washing step, cells were incubated with goat anti-mouse Cy3 antibody (1:400, Jackson ImmunoResearch) for 1 hour at RT. Samples were washed with PBS and nuclei were stained with 1 μg/ml Hoechst (Sigma-Aldrich). Coverslips were finally mounted on glass slides (glycerol PBS 9:1, pH = 8.5–9.0) and image acquisitions were taken by Nikon 90i wide-field fluorescence microscope. RAW images were processed into TIF files using ImageJ open-source software.

#### Electrophysiological Experiments

##### Electrophysiology

HEK-293 cells were grown in DMEM supplemented with 10% heat-inactivated FBS and 1% penicillin-streptomycin, at 37°C in a 5% CO_2_ humidified atmosphere. Cells were plated on cover slides (8 x 10^4^ cells/ml) and, after 24 h, transiently transfected using Lipofectamine 3000 (*Invitrogen*) according to the manufacturer's protocol, and adding 0.5 μg of plasmid DNA subtype per well. Recordings were carried out 24–36 h following transfection. Electrophysiological experiments were performed using the whole-cell configuration of the patch-clamp technique. Recordings were obtained using a HEKA EPC800 amplifier, Digidata 1322A analog-to-digital converter, and pClamp 10 software (Molecular Devices, Union City, CA). Data were filtered at 2 kHz and digitized at 5 kHz. Normal external solution contained: 140 mM NaCl, 2.8 mM KCl, 2 mM MgCl_2_, 2 mM CaCl_2_, 10 mM HEPES, and 10 mM glucose (pH 7.4; 300 mosM). The internal pipette solution contained: 140 mM CsCl, 5 mM BAPTA, 2 mM Mg-ATP, and 10 mM HEPES (pH 7.4; 300 mOsm). Borosilicate glass pipettes were pulled with a Narishige puller to a typical pipette resistance of 3–4 MΩ. Cell capacitance was measured for each cell and access resistance compensated to 70%.

##### Data Acquisition and Analysis

The current-voltage protocol stepped the cell membrane potential from −120 mV to test potentials starting at −110 mV and increasing to 20 mV in 10 mV increments. Test potentials were 100 ms in duration, and the membrane potential was returned to −120 mV for 10 s between acquisitions to allow complete recovery from inactivation. Peak inward Ca^2+^ currents were plotted as a function of the test potential to generate current-voltage relations (I-V). The peak currents were also normalized by the individual cell capacitance measurement for the comparison of current densities. Mean current density-V relations were fit with a modified form of the Boltzmann equation, where Ipeak = (V – Erev) Gmax (1/1 + exp (Vh – V)/S)), and Erev is the reversal potential, Vh is the half-activation potential, Gmax is the maximum slope conductance, and S is the slope factor that is inversely proportional to the effective gating charge. To assess the voltage dependence of inactivation, the cell membrane was stepped from a holding potential of −120 mV to conditioning potentials 1 s in duration between −120 and −60 mV in 10 mV increments before proceeding to a test potential of −40 mV for 100 ms, from which the resulting inward Ca^2+^ currents were analyzed. The voltage of half-inactivation (Vi) was estimated from Boltzmann fits of I/Imax vs. voltage where I/Imax = 1/(1 + exp (z^*^(V – Vi)/25.6)). Clampfit 10 was used to analyse all data obtained in Clampex (Molecular Devices). Current kinetics were evaluated at −40 mV test potential by measuring the time from basal to peak current, and by fitting the current decay with a single exponential equation. Fits of the I-V relations, activation and inactivation curves, and decays were carried out in SigmaPlot (Jandel Scientific). Data are presented as the means ± S.E. Statistical tests was done with one-way analysis of variance (ANOVA).

## Results

### Identification of *CACNA1H* Biallelic Variants From WGS

We performed WGS analysis in a cohort of 105 ASD families, including 124 ASD individuals, 210 parents and 58 unaffected siblings. Within WGS data, we explored the presence of rare coding damaging variants in voltage-gated calcium channels (VGCCs) genes, to investigate their role in ASD development in the affected individuals of our cohort. Specifically, we looked for damaging variants with MAF ≤ 0.1% (ultra-rare variants) that could potentially act under a dominant model of inheritance and variants meeting a less stringent threshold of MAF ≤ 1% (rare variants), possibly acting under a recessive inheritance model (homozygous, hemizygous and compound heterozygous variants).

We identified 53 ultra-rare damaging variants in 17 VGCCs genes in 41 ASD individuals (Supplementary Table 1). All these variants were inherited from unaffected parents and no *de novo* variants were identified. Among recessive-acting variants, 2 hemizygous variants in *CACNA1F* and 4 compound heterozygous variants in *CACNA1H* emerged from our analysis (Table 1). The presence of recessive-acting mutations in two VGCCs genes led us to evaluate the hypothesis that recessive model could be a shared mechanism of action of variants affecting voltage-gated calcium channels. Given the classification of *CACNA1H* as a strong ASD candidate gene in the SFARI Gene database (SFARI score = 2, https://gene.sfari.org/database/human-gene/CACNA1H), we decided to further investigate the role of biallelic variants in this gene.

**Table 1:** Rare recessive-acting damaging variants in VGCCs genes identified in our WGS data set.

**Proband ID (sex)**	**Gene (SFARI score)**	**Genomic change (hg38)**	**Amino acid change**	**CADD score**	**Inheritance**	**dbSNP**	**Total MAF (gnomAD v.3.0)**
105.3 (M)	*CACNA1H* (2)	NC_000016.10: g.1204361A>T	NP_066921.2: p.(Lys785Met)	24.7	Maternal	rs28365117	0.00300000
		NC_000016.10: g.1205207C>T	NP_066921.2: p.(Pro849Ser)	18.22	Paternal	rs370675810	0.00009770
22.3-22.4 (F-F)	*CACNA1H* (2)	NC_000016.10: g.1220303C>T	NP_066921.2: p.(Pro2124Leu)	19.03	Maternal	rs372453886	0.00006978
		NC_000016.10: g.1220945C>T	NP_066921.2: p.(Ser2338Phe)	16.99	Paternal	rs757713867	0.00002792
5.3 (M)	*CACNA1F* (3)	NC_000023.11: g.49211360C>T	NP_005174.2: p.(Ala1419Thr)	18.34	Maternal	rs782741094	0.00020000
112.3 (M)	*CACNA1F* (3)	NC_000023.11: g.49211983C>T	NP_005174.2: p.(Gly1350Ser)	24.7	Maternal	rs782780521	0.00002839

**Figure 1:**
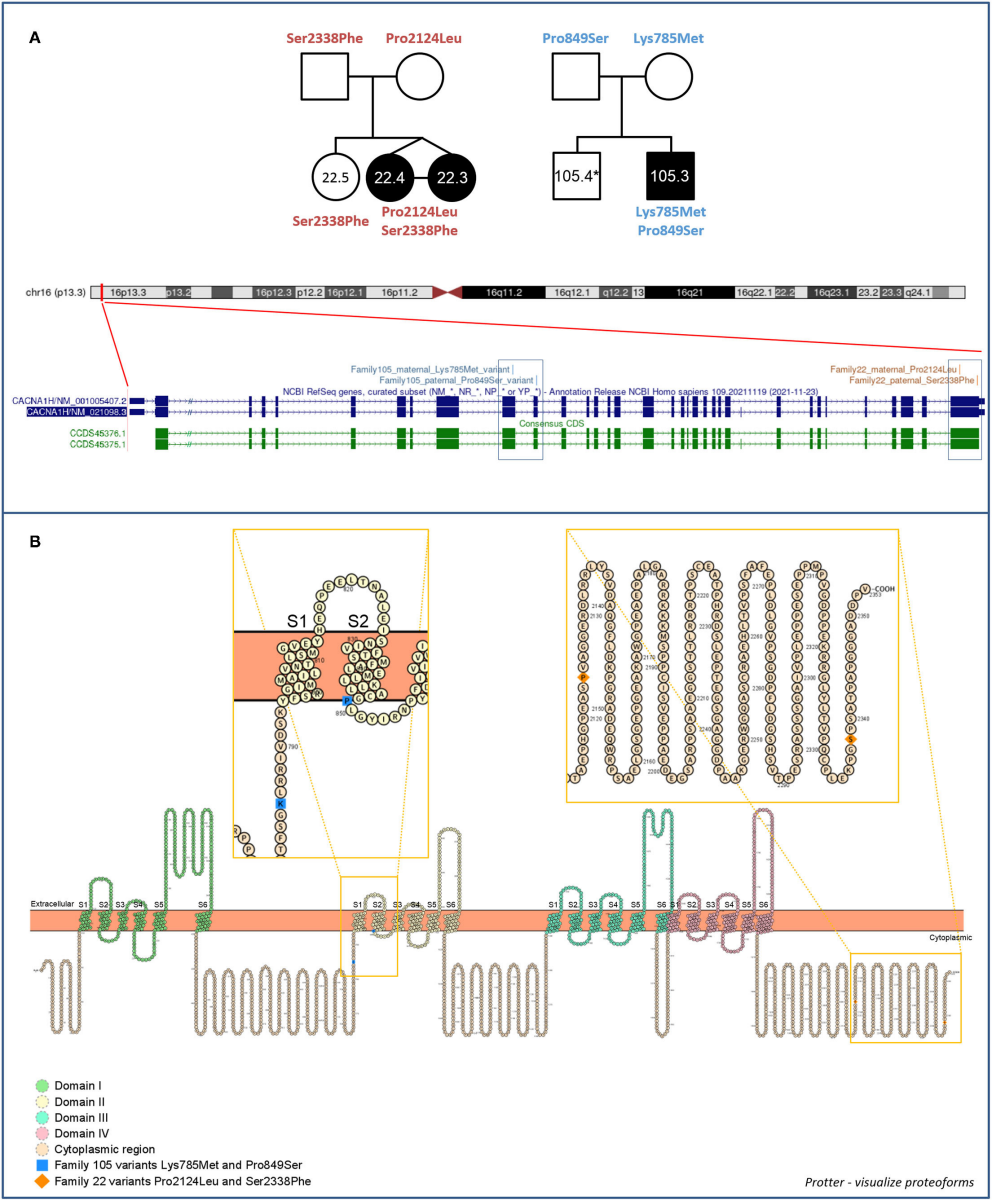
Family 22 and 105 *CACNA1H* variants. **(A)** Family segregations and gene location of *CACNA1H* biallelic variants. Filled shapes indicate ASD individuals. *DNA was not available for individual 105.4. UCSC hg38 Genome Browser screenshot shows the location of biallelic variants within the *CACNA1H* gene. **(B)** Schematic of the *CACNA1H* protein channel (Ca_v_3.2). Ca_v_3.2 channel consists of the single α1 pore-forming subunit of about 260 kDa, organized in four homologous domain each composed of six transmembrane segments (S1–S6). Within each domain, the arginine/lysine-rich S4 segment represents the voltage-sensing region of the channel, while the extracellular loop linking S5 and S6 segments (P loop) ensures the ion conductivity and selectivity of the channel (14, 18). Protein visualization was generated using Protter–visualize proteoforms (19).

Compound heterozygous missense variants in *CACNA1H* were present in 3 ASD individuals belonging to 2 unrelated ASD families. In particular, two *CACNA1H* SNVs, one inherited from the father and one inherited from the mother, were identified in two female monozygotic ASD twins (indicated as proband 22.3 and proband 22.4), and other two *CACNA1H* variants, one paternally and one maternally inherited, were identified in the male proband of a trio (indicated as proband 105.3) (Figure 1A).

All identified variants were rare coding non-synonymous variants predicted to be damaging for the normal protein function (CADD score ≥15) (16) (Table 1). Proband 105.3 paternal and maternal variants are located within *CACNA1H* exons 10 and 11, respectively. The maternal substitution A > T causes the substitution of a lysine with methionine (NP_066921.2:p.Lys785Met) in the cytoplasmic loop linking protein domains I and II; the paternal change C > T affects the last amino acid of the transmembrane segment 2 of the protein domain II, leading to the substitution of proline 849 with a serine (NP_066921.2:p.Pro849Ser) (Figure 1B). The two non-synonymous variants identified in the twins (22.3 and 22.4) are located in the last exon of the gene (exon 35), causing respectively the amino acid change Pro2124Leu and Ser2338Phe (NP_066921.2) in the cytoplasmic C-terminal region of the protein (Figure 1B). The biallelic condition was not shared with the unaffected sister (22.5), who inherited only the paternal variant (Figure 1A).

In both families, no clear pathogenic sequence variants were identified from WGS analysis. Among ultra-rare (MAF ≤ 0.1%) variants of uncertain significance (VUS) emerged from WGS analysis in the two families, the most interesting variants were one *de novo* missense variant in *PRSS2* and 9 inherited missense variants predicted to be damaging in SFARI genes (SFARI score 2 and 3) in proband 105.3, and one *de novo* novel missense variant in *ARFGEF3* and 20 inherited ultra-rare potentially deleterious variants (2 LGD and 18 damaging missense variants) in SFARI genes (with SFARI score 1, 2 and 3) in probands 22.3 and 22.4 (Supplementary Table 2).

Additional heterozygous deleterious ultra-rare variants in *CACNA1H* were identified in other 9 ASD individuals of our cohort, one of them was also shared with an unaffected sister (Supplementary Table 1). No biallelic variants in *CACNA1H* were detected in parents or unaffected siblings of our sample.

### Clinical Characterization of ASD Individuals Carrying *CACNA1H* Biallelic Variants

The two probands of family 22 are 9.9-year-old monozygotic female twins, born from consanguineous parents coming from Bangladesh. Family history is positive for ASD in one maternal first cousin. Twins were born at 33 weeks of pregnancy through cesarean delivery due to premature rupture of membrane. They were hospitalized due to prematurity, respiratory distress, hyperbilirubinemia, and feeding problems. Motor and language development were delayed for both. They showed early-onset atypical socio-communicative skills, restricted/repetitive interests and activities, and sensory abnormalities. Both twins were diagnosed with ASD at the age of 40 months using ADOS-2 [severity level 3, according to DSM-5 (2)]. Twin 22.3 had 4 febrile convulsions (from 18 months to 5 years of age), followed by two apparently generalized convulsive seizures without fever (the last one was a status epilepticus). EEG showed focal (right mid-posterior) and diffuse paroxysmal abnormalities. Seizures remitted with topiramate treatment. Twin 22.4 had no seizures and her EEG was normal. Intellectual disability was present in both twins: severe for twin 22.3 and moderate for twin 22.4. Neurological examination showed lack of speech and stereotypies in both. Array-CGH showed no pathogenetic copy number variants. Brain MRI (1,5 Tesla) was normal for both twins.

The proband of family 105 is a 8.5-year-old boy, born from non-consanguineous Italian parents. Family history is positive for learning disability in one paternal first cousin. Pregnancy, delivery and neonatal period were normal. Motor development milestones were acquired regularly, while language was delayed. At 1 year of age, social communication deficits and restricted/repetitive interests and activities as well as sensory abnormalities became evident. At 40 months of age, he was diagnosed with ASD through ADOS-2 [severity level 3, according to DSM-5 (2)]. A moderate intellectual disability was associated. Neurological examination showed speech delay and stereotypies with upper and lower arms. Array-CGH showed no pathogenic copy number variants. Molecular analysis for fragile X syndrome was negative. EEG showed frequent multifocal paroxysmal abnormalities (spike-waves) in the left central and in the right centro-temporal regions, slightly increased in the early stages of sleep. The boy never presented epileptic seizures. Brain MRI (1,5 Tesla) was normal. Additional clinical data are reported in Supplementary Table 3.

### Functional Characterization of *CACNA1H* Biallelic Variants

In order to perform functional analysis to clarify the effect of the *CACNA1H* variants on the calcium channel activity, we generated 3xFLAG-Ca_v_3.2 wild-type (WT) and 4 3xFLAG-Ca_v_3.2 mutant recombinant proteins, corresponding to paternal and maternal mutations identified in families 22 and 105.

**Figure 2:**
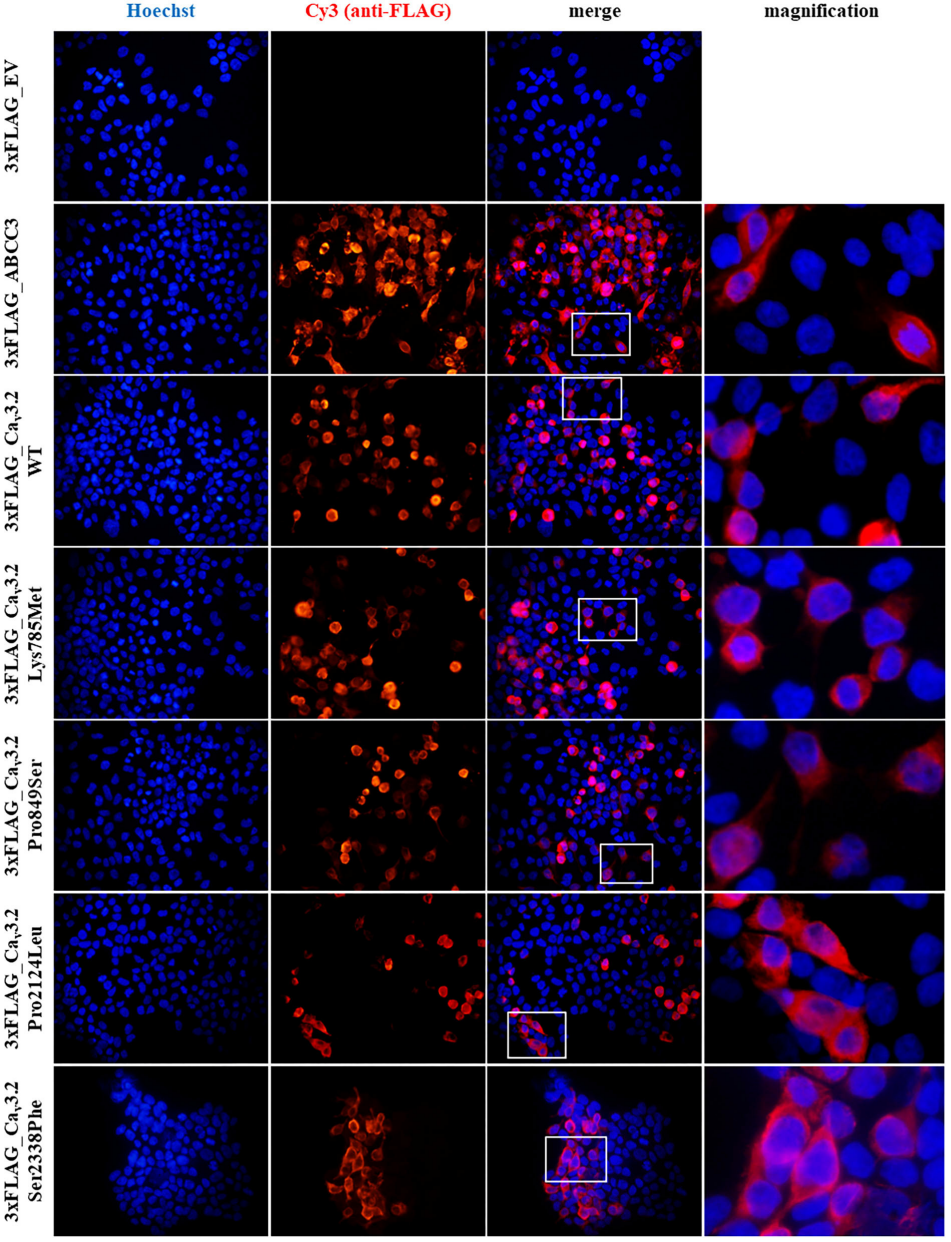
Immunofluorescence (IF) assay of 3xFLAG-Ca_v_3.2. IF assay was performed in HEK-293T cells, by transiently transfecting cells with p3xFLAG-CACNA1H plasmid constructs. An empty vector (EV) was used as negative control, while 3xFLAG-ABCC3 transmembrane fusion protein was used as positive control. Mouse anti-FLAG M2 antibody was used for detection of the recombinant proteins. Goat anti-mouse Cy3 antibody (red signal) and Hoechst dye (blue signal) were employed to detect anti-FLAG antibody and nuclei respectively.

Recombinant proteins expression in mammalian cells and their cellular localization were assessed by immunofluorescence (IF) assay in HEK-293T cells. As illustrated in Figure 2, by merging Hoechst and Cy3 signals, indicating nuclei and recombinant proteins respectively, WT and mutant recombinant proteins were expressed in our cell system and correctly localize at the cell membrane, with no considerable differences in cellular localization among them and compared to the positive control (Figure 2).

**Figure 3:**
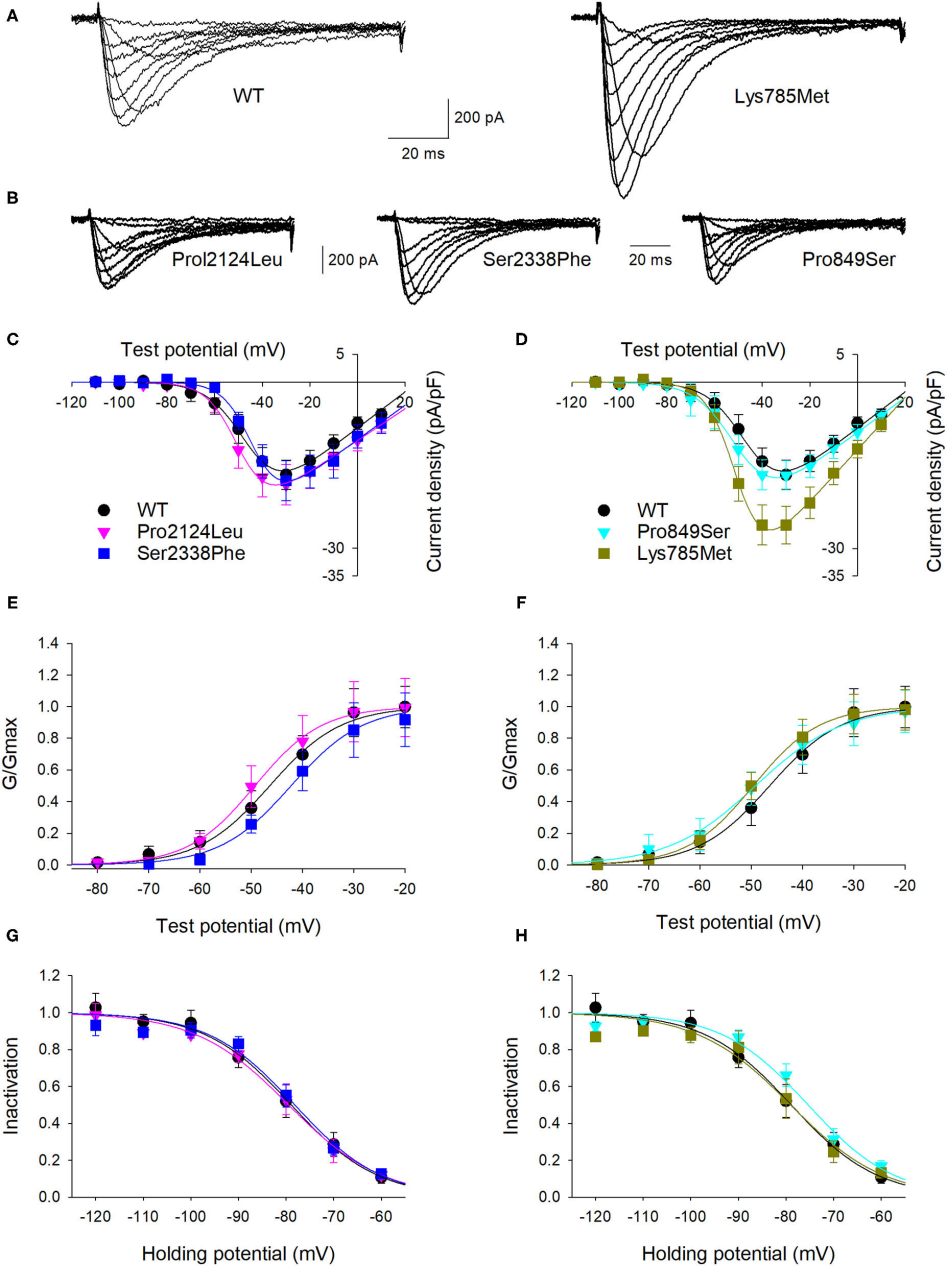
Lys785Met is a gain of function Ca_v_3.2 subtype. **(A)** Representative current traces recorded from two HEK-293 cells transiently transfected with the WT (left) or Lys785Met (right) CACNA1H plasmid construct. The currents were elicited by step depolarizations from a holding potential of −120 mV to various test potentials. **(B)** Representative current traces recorded from three HEK-293 cells transiently transfected with the Pro2124Leu (left) or Ser2338Phe (middle) or Pro849Ser (right) plasmid construct, same protocol as **(A)**. **(C)** Mean activation curves for WT, Pro2124Leu and Ser2338Phe plasmid constructs transfected cells, as indicated. Solid lines represent data fit to the Boltzmann equation (Vh values are −45.5, −49.3 and −43.4 mV for WT, Pro2124Leu and Ser2338Phe, respectively). Data were averaged from 16, 19 and 17 cells, for WT, Pro2124Leu and Ser2338Phe, respectively. **(D)** Mean activation curves for WT (same data as **C**), Pro849Ser and Lys785Met plasmid constructs transfected cells, as indicated. Solid lines represent data fit to the Boltzmann equation (Vh values are −45.5, −49.5 and −50.1 mV for WT, Pro849Ser and Lys785Met, respectively). Data were averaged from 16 (same cells as **C**), 16 and 14 cells, for WT, Pro849Ser and Lys785Met, respectively. **(E)** Normalized mean activation curves for WT, Pro2124Leu and Ser2338Phe plasmid constructs transfected cells, same data as **(C)**. Solid lines represent data fit to the activation Boltzmann equation. **(F)** Normalized mean activation curves for WT, Pro849Ser and Lys785Met plasmid constructs transfected cells, same data as **(D)**. Solid lines represent data fit to the activation Boltzmann equation. **(G)** Inactivation open probability-voltage relationships for WT, Pro2124Leu and Ser2338Phe plasmid constructs transfected cells. Solid lines represent data fit to the inactivation Boltzmann equation (Vi values are −78.9, −79.5 and −78.2 for WT, Pro2124Leu and Ser2338Phe, respectively). No significant differences were detected. **(H)** Inactivation open probability-voltage relationships for WT, Pro849Ser and Lys785Met plasmid constructs transfected cells. Solid lines represent data fit to the inactivation Boltzmann equation (Vi values are −78.9, −75.3 and −77.1 mV for WT, Pro849Ser and Lys785Met, respectively). No significant differences were detected.

**Figure 4:**
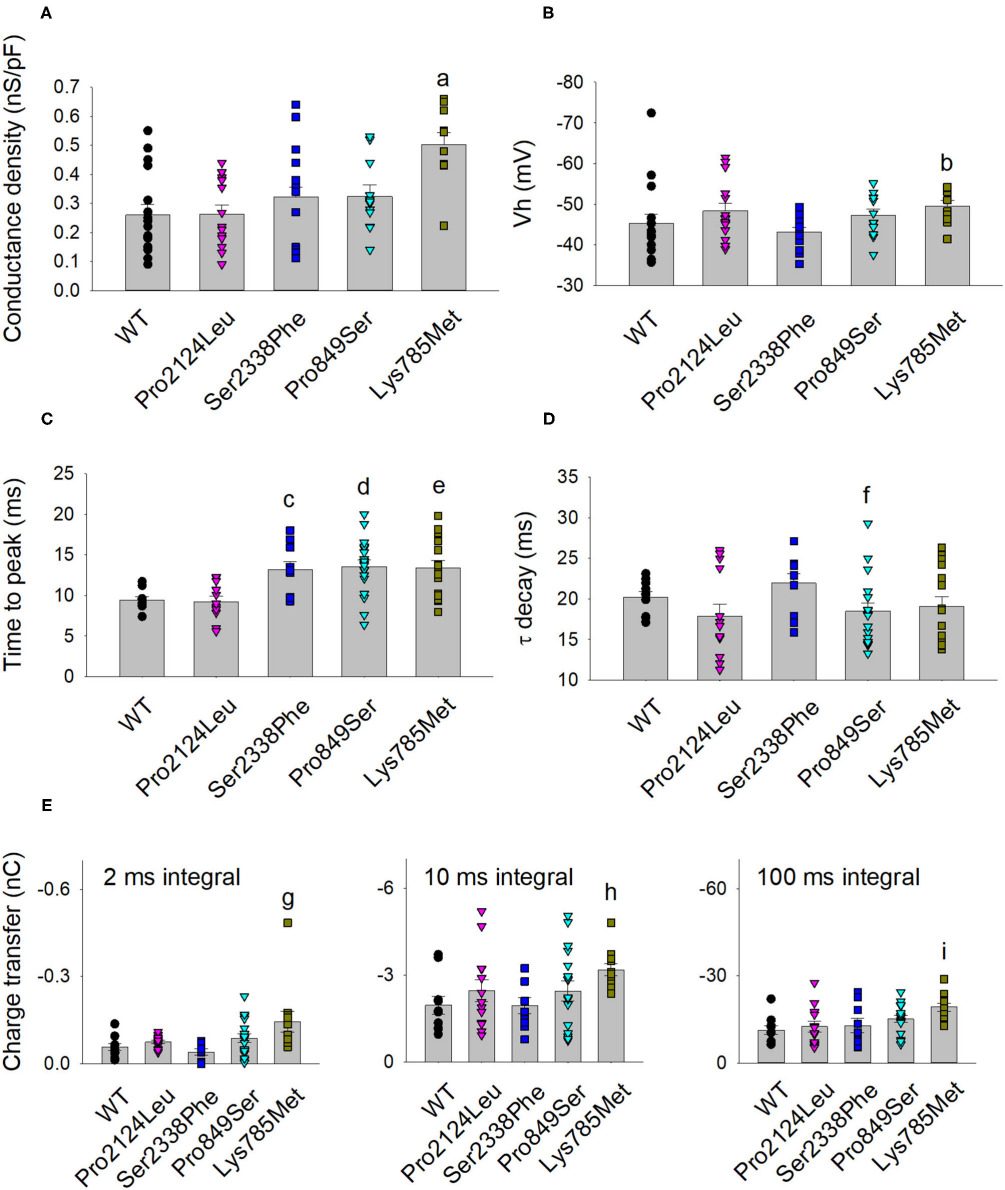
Activation and kinetic parameters of Lys785Met, Ser2338Phe and Pro849Ser Ca_v_3.2 subtypes are different from WT. **(A)** Histogram representing the mean conductance density of WT and mutant channels expressed in HEK-293 cells, measured at −40 mV test potential for each Ca_v_3.2 subtype, as indicated. Mean values were averaged from 16, 15, 13, 12 and 10 cells, from left to right. The conductance density was significantly higher for Lys785Met mutant, as compared to WT (a, *p* < 0.001). **(B)** Histogram representing the mean Vh value measured at −40 mV test potential for each Ca_v_3.2 subtype, as indicated. Same cells as **(A)**. The Vh value was significantly higher for Lys785Met mutant, as compared to WT (b, *p* = 0.037). **(C)** Left, histogram representing the mean values of time to peak measured at −40 mV test potential for each Ca_v_3.2 subtype, as indicated. Mean values were averaged from 10, 13, 10, 18 and 15 cells, from left to right. The time to peak was significantly higher for Ser2338Phe, Pro849Ser and Lys785Met mutants, as compared to WT (c, *p* = 0.002; d, *p* = 0.002; e, *p* = 0.003). **(D)** Histogram representing the mean values of exponential τ decay measured at −40 mV test potential for each Ca_v_3.2 subtype, as indicated. Same cells as **(C)**. The time to peak was significantly higher for Pro849Ser mutant, as compared to WT (f, *p* = 0.039). **(E)** Histograms representing the mean values of charge transfer measured at −40 mV test potential for each Ca_v_3.2 subtype, as indicated, at different current times (left, 2 ms; center, 10 ms; right, 100 ms). Same cells as **(C)**. The charge transfer value was significantly higher only for Lys785Met mutant, at each time point, as compared to WT (g, *p* = 0.004; h, *p* = 0.010; i, *p* < 0.001).

To highlight possible functional effects due to identified SNVs, electrophysiological experiments were performed using the whole-cell patch-clamp technique. Plasmid encoding mutant 3xFLAG-Ca_v_3.2, along with plasmid encoding the WT isoform, were transfected in HEK-293 cells, and the resulting voltage-activated Ca^2+^ currents were recorded and analyzed. Cells transfected with plasmid DNA coding for the mutant proteins exhibited inward currents in response to depolarization steps, similarly to WT protein (Figures 3A,B). Loss of function was not observed in any mutant channel, while current densities (Figures 3C,D) and activation properties (Figures 3E,F) were differently modulated by distinct mutations. Specifically, cells transfected with plasmid DNA encoding the Lys785Met-mutant Ca_v_3.2 exhibited a larger mean current density (Figures 3D, 4A), and a left shift in the activation I-V curves, with a more hyperpolarized Vh value of −50 ± 1 mV, compared to the WT Vh value of −45 ± 2 mV (Figure 4B; *p* = 0.037). The inactivation properties were unaffected by the mutations (Figures 3G,H).

The kinetics of inward currents, elicited by a −40 mV depolarization step, were differently affected by the mutations: in comparison with the WT values, currents mediated by the Ser2338Phe, Pro849Ser and Lys785Met mutant channels showed a slower time to peak (Figure 4C), and those mediated by the Pro849Ser isoform also decayed more rapidly (Figure 4D). To evaluate the impact of these altered parameters on the channel function, we measured the total charge transfer (i.e., the total amount of Ca^2+^ ions entering the cells) induced by a −40 mV depolarization step, at different time points (2, 10 and 100 ms): a significant increase was observed at all time points only in cells transfected with the Lys785Met -mutant Ca_v_3.2 expression plasmid (Figure 4E).

## Discussion

Voltage-gated calcium channels (VGCCs or Ca_v_ channels) are transmembrane protein mediating calcium ions influx into excitable cells upon depolarization of the cell membrane. The family of VGCCs includes distinct types of channels, differing in electrophysiological properties. High-voltage-activated (HVA) calcium channels consist of Ca_v_1 and Ca_v_2 subfamilies, while low-voltage-activated (LVA) calcium channels consist of Ca_v_3 subfamily exclusively. The principal functional subunit of Ca_v_ channel is the pore-forming α1 subunit, encoded by the *CACNA1A* to *CACNA1I* and *CACNA1S* genes. A single α1 subunit constitutes the functional form of LVA or Ca_v_3 channels, while HVA (Ca_v_1 and Ca_v_2) channels require the presence of α_2_δ and β auxiliary subunits, encoded by the four *CACNA2D1-4* genes and the four *CACNB1-4* genes respectively. The α1 subunit co-assemblies with one of four α_2_δ and one of four β subunits, forming HVA multiprotein functional complex (20, 21). Even if the main properties of the channel are determined by the pore-forming unit both in LVA and HVA channels, in the latter group biophysical properties and the pore-forming unit targeting at the cell membrane are profoundly modulated by the auxiliary subunits (13, 22–24).

By mediating Ca^2+^ entry, VGCCs are involved in multiple processes critical for cellular function, thus their dysfunction is associated with a wide range of different diseases, including neuropsychiatric disorders. VGCCs have been consistently implicated in schizophrenia, ADHD, ASD, epilepsy, bipolar disorder, anxiety and major depressive disorder (MDD), implicating Ca_v_ channels altered function in dysregulation of calcium signaling, postsynaptic function, synaptic plasticity and gene transcription (10, 21, 25, 26). Specifically, several studies investigated the role of both rare and common variants in the α1 and the auxiliary subunits of VGCCs genes in ASD, highlighting the implication of voltage-dependent calcium channels in the disease susceptibility (12). Moreover, 13 out of 18 VGCCs genes are included in the SFARI Gene database (https://gene.sfari.org/), a curated list of genes implicated in ASD susceptibility. Specifically, 4 VGCCs genes (*CACNA1A, CACNA1C, CACNA1E* and *CACNA2D3*) are reported to be clearly implicated in ASD (SFARI 1), while other 3 genes (*CACNA1D, CACNA1H* and *CACNB2*) are defined as strong ASD candidate genes (SFARI 2).

In order to investigate the role of Ca_v_ channels in ASD etiology, we looked for rare coding damaging variants in VGCCs genes in WGS data of 105 ASD families, which include 124 ASD individuals. We identified 53 ultra-rare damaging variants in VGCCs genes, none of them was *de novo*. Interestingly, about one third of ASD individuals of this cohort (41 out of 124) had a deleterious variant in at least one VGCCs gene, with 10 ASD individuals carrying deleterious variants in two or more VGCCs genes. Moreover, four rare damaging biallelic variants were detected in the *CACNA1H* gene in two ASD families.

No biallelic variants in *CACNA1H* have been previously reported in individuals with ASD. However, *CACNA1H* compound heterozygous variants have been previously identified in neuromuscular disorders, for which functional analysis showed mild but significant changes on T-type channel activity that are consistent with a loss of channel function (27–29).

The *CACNA1H* gene encodes the LVA T-type calcium channel Ca_v_3.2, that is widely distributed in excitable cells, including brain where it is highly expressed in thalamus, hippocampus, amygdala and putamen. Ca_v_3.2 regulates intracellular calcium concentration, playing important roles in neuronal firing and in neurotransmitter release (14, 30, 31).

*CACNA1H* is reported to be a strong candidate for ASD as both *de novo* and inherited rare variants in *CACNA1H* were identified in individuals with ASD and other neurodevelopmental disorders (15, 32–40), and functional analysis showed a significant effect of *CACNA1H* variants on channel function. Specifically, Splawski et al. tested five missense variants identified in a sample of 461 ASD individuals, and detected a decreased activity in mutant channels, implicating *CACNA1H* missense variants in ASD risk with loss-of-function mechanism and incomplete penetrance (15).

We identified biallelic variants in the *CACNA1H* gene in 3 ASD individuals (2 monozygotic twins and an unrelated proband) from two families. Probands 22.3-22.4 biallelic variants (Ser2338Phe and Pro2124Leu) affect the cytoplasmic C-terminal domain of the channel, not directly implicated in ion transport and channel functionality but with a putative regulatory role by interacting with Sintaxin-1A (41); proband 105.3 variants (Pro849Ser and Lys785Met) are located in protein regions expected to have a greater effect on the T-type calcium channel function. Indeed, several variants affecting the same channel region were previously identified and functionally tested, with some of them showing a functional effect (40). Specifically, proband 105.3 paternal Pro849Ser variant is located in the S2 segment of the second protein domain, in which two gain of function mutations were already identified in individuals with Childhood Absence Epilepsy (42, 43). Moreover, the maternal Lys785Met mutation is located in the cytosolic I-II loop, that is an important regulator of channel function, contributing to the regulation of its gating properties and surface expression (44, 45). Several variants were identified in this channel region in individuals affected by idiopathic generalized epilepsy, neuromuscular disorder and chronic pain, with most of them occurring in the region surrounding Lys785Met variant, some of which showing gain of function effect (40).

We functionally characterized these four *CACNA1H* variants to clarify their effect on the Ca_v_3.2 channel activity. No decrease in the voltage-gated Ca^2+^ conductance was registered for any of the tested variants, thus excluding loss of function effects. In contrast, a clear gain of function effect was observed for the Lys785Met mutant channel, which exhibited a higher functional expression in HEK293 cells, along with a significantly more hyperpolarized Vh value, indicating a higher open probability than WT at the same potential. These properties conferred the ability to transfer, upon activation, a larger electrical charge to the Lys785Met mutant channel when compared to WT, suggesting an increased Ca^2+^ entry in neurons expressing this mutant channel. A detailed kinetic analysis revealed that three out of four mutants (Ser2338Phe, Pro849Ser and Lys785Met) exhibited altered activation kinetics. Therefore, in family 22 only one *CACNA1H* variant caused mild alterations in channel properties, while both variants in family 105 showed a functional effect, even if with variable intensity and acting on different channel properties. It is thus likely that the identified variants in *CACNA1H* may lead to subtle dysregulation of intracellular Ca^2+^ concentration, thereby altering neuronal cell signaling and gene transcription. Our results suggest that *CACNA1H* variants influence the ASD phenotype with incomplete penetrance, driving individual susceptibility over the ASD threshold, together with other risk variants. Indeed, in both families the probands inherited additional rare damaging variants in other high-confidence ASD genes. Since only one of the twins of family 22 had fever triggered epilepsy, we might hypothesize that *CACNA1H* variants could also contribute to epilepsy, but with variable penetrance.

In conclusion, in the present study we investigated the involvement of VGCCs rare variants in ASD susceptibility. This led to the identification of *CACNA1H* biallelic mutations in probands from two families. Functional characterization by patch-clamp revealed that three out of four tested variants cause mild alterations of the Ca_v_3.2 channel properties. Our results provide additional support to previous studies implicating *CACNA1H* in neurodevelopmental disorders and suggest that *CACNA1H* mutations may be involved in the pathophysiology of ASD.

## Data Availability Statement

The datasets that support the findings of this study can be found in the Supplementary Material of this article. Requests to access the raw data should be directed to elena.bacchelli@unibo.it.

## Ethics Statement

The studies involving human participants were reviewed and approved by Comitato Etico di Area Vasta Emilia Centro (CE-AVEC); code CE 14060. Written informed consent to participate in this study was provided by the participants' legal guardian/next of kin. Written informed consent was obtained from the individual(s), and minor(s)' legal guardian/next of kin, for the publication of any potentially identifiable images or data included in this article.

## Author Contributions

MV participated to study design, performed WGS data bioinformatic analysis, performed cloning experiments, immunofluorescence assay, wrote the manuscript, and prepared figures and tables. TD'A performed electrophysiological analyses and contributed to figures preparation. CC participated to study design and contributed to WGS data bioinformatic analysis. AP, PV, MS, RC, and MR collected the samples and performed the clinical characterization of patients. FC participated to study design and contributed to manuscript writing. GM designed and supervised cloning experiments and immunofluorescence assay. SF supervised electrophysiological, data analyses and contributed to manuscript writing. EM participated to study design, supervised all analyses and contributed to manuscript writing. EB participated to study design, performed WGS data bioinformatic analysis, supervised all analyses and contributed to manuscript writing. All authors read and approved the final manuscript.

## Funding

This research was funded by Italian Ministry of Health, grant number GR-2013-02357561, and by RFO (University of Bologna). WGS data were generated at the New York Genome Center with funds provided by NHGRI Grant 3UM1HG008901. The Centers for Common Disease Genomics are funded by the National Human Genome Research Institute and the National Heart, Lung, and Blood Institute.

## Conflict of Interest

The authors declare that the research was conducted in the absence of any commercial or financial relationships that could be construed as a potential conflict of interest.

## Publisher's Note

All claims expressed in this article are solely those of the authors and do not necessarily represent those of their affiliated organizations, or those of the publisher, the editors and the reviewers. Any product that may be evaluated in this article, or claim that may be made by its manufacturer, is not guaranteed or endorsed by the publisher.
