# Supplementary material for: Contribution of CACNA1H Variants in Autism Spectrum Disorder Susceptibility
Source: Front Psychiatry. 2022 Mar 8;13:858238. doi: 10.3389/fpsyt.2022.858238 (PMC8957782; doi:10.3389/fpsyt.2022.858238)
Supplement: Supplementary file 1 [file Data-Sheet_1.pdf]

Supplementary Material

1 Supplementary Data

Sequence of primers employed for site-directed mutagenesis through whole-plasmid PCR is reported below. Mismatch sites corresponding to changes introduced in plasmid constructs sequence through whole-plasmid PCR are reported in bold.

Primer name	Sequence (5'-3')
CACNA1H_K785M_F	gctgcgccgcatcgtgga
CACNA1H_K785M_R	**a**tgccgctgaaggtaacccagag
CACNA1H_P849S_F	gc**t**ctctgggctacatccggaac
CACNA1H_P849S_R	cgcaggccagcagcttca
CACNA1H_P2124L_F	ggtggccggcggcgagc
CACNA1H_P2124L_R	**a**gagaggcttcggggccatg
CACNA1H_S2338F_F	cccctcagccacccctg
CACNA1H_S2338F_R	**a**accctggtttctccagaggac

2 Supplementary Tables

Supplementary Table 1. Ultra-rare deleterious variants in VGCCs genes identified in our WGS data set. ASD affected individuals are indicated in bold.

Genomic change (hg38)	Amino acid change	Effect (CADD score)	Individual ID (Inheritance)	dbSNP	Total MAF (gnomAD v.3.0)	Gene	SFARI Gene score
NC_000002.12:g. 151860740A>G	NP_001005747.1: p.(Ile246Thr)	missense (27.4)	**98.3** (Paternal)	rs765961368	0.00000698	*CACNB4*	.
NC_000009.12:g. 138023642C>T	NP_000709.1: p.(Arg967Trp)	missense (18)	**66.3** (Maternal)	rs200418906	0.00004250	*CACNA1B*	3
NC_000009.12:g. 138121659G>A	NP_000709.1: p.(Arg2227Gln)	missense (24.6)	**31.3**-31.4 (Paternal)	.	0.00000698	*CACNA1B*	3
NC_000009.12:g. 138121688G>A	NP_000709.1: p.(Ala2237Thr)	missense (15.73)	**87.3**-87.4 (Maternal)	rs77641565	0.00030000	*CACNA1B*	3
NC_000009.12:g. 138121809C>T	NP_000709.1: p.(Thr2277Ile)	missense (22.9)	**60.3** (Paternal)	rs760115118	0.00006980	*CACNA1B*	3
NC_000009.12:g. 138121907G>A	NP_000709.1: p.(Val2310Met)	missense (22.4)	**18.3** (Paternal)	rs201689533	0.00002090	*CACNA1B*	3
NC_000023.11:g. 49211360C>T	NP_005174.2: p.(Ala1419Thr)	missense (18.34)	**5.3** (Maternal)	rs782741094	0.00020000	*CACNA1F*	3
NC_000023.11:g. 49211983C>T	NP_005174.2: p.(Gly1350Ser)	missense (24.7)	**112.3** (Maternal)	rs782780521	0.00002840	*CACNA1F*	3

NC_000001.11:g. 181790533C>A	NP_001192222.1: p.(Gln1959Lys)	missense (16.89)	**43.3** (Paternal)	rs761958333	.	*CACNA1E*	1
NC_000001.11:g. 201048619_2010 48620del	NP_000060.2: p.(Phe1468Cysfs*14)	frameshift deletion (.)	**40.4** (Maternal)	.	.	*CACNA1S*	.
NC_000001.11:g. 201051096T>C	NP_000060.2: p.(Tyr1334Cys)	missense (25)	**46.3**-46.4 (Maternal)	rs146158332	0.00006980	*CACNA1S*	.
NC_000001.11:g. 201062475C>G	NP_000060.2: p.(Glu965Gln)	missense (21)	**49.3** (Maternal)	rs762350071	0.00004890	*CACNA1S*	.
NC_000001.11:g. 201075539A>G	NP_000060.2: p.(Met635Thr)	missense (23.2)	**14.4** (Paternal)	rs144590408	0.00002800	*CACNA1S*	.
NC_000003.12:g. 50366093C>T	NP_001005505.1: p.(Arg927His)	missense (23.3)	**101.3** (Maternal)	rs761523126	0.00003490	*CACNA2D2*	.
NC_000003.12:g. 53775936G>A	NP_000711.1: p.(Gly1438Glu)	missense (26.4)	**54.3** (Paternal)	.	.	*CACNA1D*	2
NC_000003.12:g. 54627841A>G	NP_060868.2: p.(Ile340Val)	missense (19.62)	**54.3**-54.4 (Maternal)	rs185055678	0.00009770	*CACNA2D3*	1
NC_000003.12:g. 54764331G>A	NP_060868.2: p.(Glu454Lys)	missense (26.6)	**103.3** (Maternal)	rs780345468	0.00001400	*CACNA2D3*	1
NC_000003.12:g. 55018224C>T	NP_060868.2: p.(Thr965Ile)	missense (22.4)	**68.3-68.4-**68.5 (Paternal)	.	0.00000698	*CACNA2D3*	1
NC_000007.14g. 81967601C>G	NP_000713.2: p.(Asp820His)	missense (22.3)	**54.3**-54.4 (Maternal)	.	.	*CACNA2D1*	3
NC_000010.11g. 18514277G>C	NP_000715.2: p.(Aap183His)	missense (28.5)	**103.3** (Maternal)	.	.	*CACNB2*	2
NC_000010.11g. 18514515G>T	NP_963887.2: p.(Lys225Asn)	missense (22.1)	**109.3** (Maternal)	rs199539261	0.00030000	*CACNB2*	2
NC_000010.11g. 18538234C>T	NP_000715.2: p.(Leu398Phe)	missense (24.7)	**109.3** (Maternal)	rs145638628	0.00030000	*CACNB2*	2
NC_000010.11g. 18539558G>C	NP_000715.2: p.(Arg551Pro)	missense (25.2)	**56.3** (Paternal)	rs577739840	0.00008410	*CACNB2*	2
NC_000012.12:g. 1793753C>T	NP_758952.4: p.(Ala1106Thr)	missense (24.5)	**22.3-22.4** (Paternal)	.	.	*CACNA2D4*	.
NC_000012.12:g. 1854027G>A	NP_758952.4: p.(Arg724Trp)	missense (32)	**12.5** (Paternal)	rs371178386	0.00010000	*CACNA2D4*	.
NC_000012.12:g. 1875298T>C	NP_758952.4: p.(Asn587Asp)	missense (28.3)	**99.4** (Maternal)	rs759153873	.	*CACNA2D4*	.
NC_000012.12:g. 1886224G>A	NP_758952.4: p.(Ala331Val)	missense (32)	**22.3-22.4** (Paternal)	.	0.00001400	*CACNA2D4*	.
NC_000012.12:g. 1913025G>A	NP_758952.4: p.(Gln142Ter)	stopgain (34)	**60.3** (Paternal)	rs776189698	.	*CACNA2D4*	.
NC_000012.12:g. 2493262C>T	NP_955630.3: p.(Thr330Met)	missense (23.1)	**25.3** (Maternal)	rs377345545	0.00002090	*CACNA1C*	1
NC_000012.12:g. 2679514C>G	NP_955630.3: p.(Thr1769Ser)	missense (20.1)	**16.3** (Maternal)	rs554737427	0.00002090	*CACNA1C*	1
NC_000016.10:g. 1202195T>C	NP_066921.2: p.(Ile582Thr)	missense (19.97)	**60.3** (Paternal)	.	0.00000698	*CACNA1H*	2
NC_000016.10:g. 1204336C>T	NP_066921.2: p.(Arg777Cys)	missense (19.6)	**45.3** (Paternal)	rs375325893	0.00020000	*CACNA1H*	2
NC_000016.10:g. 1205117G>A	NP_066921.2: p.(Glu819Lys)	missense (21.2)	**34.3** (Maternal)	rs375165169	0.00004190	*CACNA1H*	2
NC_000016.10:g. 1205207C>T	NP_066921.2: p.(Pro849Ser)	missense (18.22)	**105.3** (Paternal)	rs370675810	0.00009770	*CACNA1H*	2
NC_000016.10:g.	NP_066921.2:	missense	**42.3**-42.4	rs202114960	.	*CACNA1H*	2

1208193A>T	p.(Asp1112Val)	(18.84)	(Maternal)				
NC_000016.10:g. 1209251C>T	NP_066921.2: p.(Arg1195Trp)	missense (22.8)	**15.3** (Paternal)	rs576035669	0.00006980	*CACNA1H*	2
NC_000016.10:g. 1210913G>A	NP_066921.2: p.(Ala1389Thr)	missense (23.1)	**62.4** (Maternal)	rs758458100	0.00001400	*CACNA1H*	2
NC_000016.10:g. 1215066G>A	NP_066921.2: p.(Arg1675Gln)	missense (31)	**17.3** (Maternal)	rs149367557	0.00006280	*CACNA1H*	2
NC_000016.10:g. 1220176C>T	NP_066921.2: p.(Arg2082Trp)	missense (18.31)	**92.3** (Maternal)	rs771719773	0.00004190	*CACNA1H*	2
NC_000016.10:g. 1220652_1220653insG	NP_066921.2: p.(Asp2243Glyfs*17)	frameshift insertion (.)	**73.3** (Maternal)	rs757244810	0.00004900	*CACNA1H*	2
NC_000016.10:g. 1220945C>T	NP_066921.2: p.(Ser2338Phe)	missense (16.99)	**22.3-22.4**-22.5 (Paternal)	rs757713867	0.00002790	*CACNA1H*	2
NC_000017.11:g. 39175200A>T	NP_000714.3: p.(Ile597Asn)	missense (23.8)	**22.3-22.4** (Paternal)	.	.	*CACNB1*	3
NC_000017.11:g. 39187570T>C	NP_000714.3: p.(Asn108Ser)	missense (23.4)	**28.3**-28.4 (Paternal)	rs768351592	0.00001400	*CACNB1*	3
NC_000017.11:g. 50569291A>T	NP_061496.2: p.(Ile161Phe)	missense (22.5)	**48.3** (Paternal)	rs368561457	.	*CACNA1G*	3
NC_000017.11:g. 50575862G>A	NP_061496.2: p.(Arg487His)	missense (22.6)	**34.3** (Maternal)	rs373257429	0.00002090	*CACNA1G*	3
NC_000017.11:g. 50596835G>A	NP_061496.2: p.(Gly1057Asp)	missense (22.5)	**66.3** (Maternal)	.	0.00001400	*CACNA1G*	3
NC_000017.11:g. 50626104C>G	NP_061496.2: p.(Leu2163Val)	missense (16.31)	**5.3** (Paternal)	.	.	*CACNA1G*	3
NC_000019.10:g. 13208986T>C	NP_001120693.1: p.(Thr2185Ala)	missense (22.3)	**113.4**-113.5 (Unknown)	rs1028538547	0.00002100	*CACNA1A*	1-S
NC_000019.10:g. 13214241C>T	NP_001120693.1: p.(Glu1979Lys)	missense (26.8)	**94.3** (Maternal)	.	.	*CACNA1A*	1-S
NC_000019.10:g. 13214276C>T	NP_001120693.1: p.(Arg1967Gln)	missense (27.2)	**4.3** (Maternal)	rs199886234	0.00040000	*CACNA1A*	1-S
NC_000022.11:g. 39570837C>T	NP_066919.2: p.(Arg29Trp)	missense (22.6)	**16.3** (Maternal)	rs760018532	0.00004890	*CACNA1I*	3
NC_000022.11:g. 39672234G>A	NP_066919.2: p.(Met1525Ile)	missense (21.2)	**4.3** (Paternal)	rs759135846	0.00000698	*CACNA1I*	3
NC_000022.11:g. 39679369G>A	NP_066919.2: p.(Gly1773Asp)	missense (15.89)	**14.3** (Maternal)	rs958740018	0.00001400	*CACNA1I*	3

Supplementary Table 2. Relevant ultra-rare deleterious variants identified in families 22 and 105. ASD affected individuals are indicated in bold.

Genomic change (hg38)	Amino acid change	Effect (CADD score)	Individual ID (Inheritance)	dbSNP	Total MAF (gnomAD v.3.0)	Gene	SFARI Gene score
**a) Family 22**							
NC_000006.12:g. 138336377T>G	*NP_065073.3: p.(Val2142Gly)*	*missense (27.1)*	***22.3-22.4*** *(De novo)*	.	.	*ARFGEF3*	.
NC_000001.11:g. 10653490G>A	NP_001073312.1: p.(Pro856Leu)	missense (20.1)	**22.3-22.4**-22.5 (Paternal)	rs367611106	.	*CASZ1*	1
NC_000011.10:g. 119341673G>A	NP_113621.1: p.(R539C)	missense (31)	**22.3-22.4** (Paternal)	rs374823079	0.00003488	*MFRP*	2

NC_000014.9:g.102947795C>T	NP_006026.3: p.(E1153K)	missense (24.4)	**22.3-22.4** (Paternal)	rs539521711	0.00004888	*CDC42BPB*	2
NC_000016.10:g.1220945C>T	NP_066921.2: p.(Ser2338Phe)	missense (16.99)	**22.3-22.4** (Paternal)	rs757713867	0.00002792	*CACNA1H*	2
NC_000019.10:g.17203202C>T	NP_004136.2: p.(S1645Leu)	missense (22.8)	**22.3-22.4**-22.5 (Maternal)	rs762171057	0.000006979	*MYO9B*	2
NC_000005.10:g.153698985G>A	NP_000818.2: p.(R455H)	missense (26.9)	**22.3-22.4**-22.5 (Paternal)	rs759117420	0.00002094	*GRIA1*	2
NC_000009.12:g.116975233C>T	NP_054729.3: p.(Val571I)	missense (24.8)	**22.3-22.4** (Paternal)	rs752549961	0.00002094	*ASTN2*	2
NC_000001.11:g.190098059G>C	NP_950252.1: p.(Pro754A)	missense (23.8)	**22.3-22.4**-22.5 (Maternal)	rs751325612	.	*BRINP3*	3
NC_000012.12:g.123838461G>A	NP_997320.2: p.(M1518I)	missense (25.1)	**22.3-22.4**-22.5 (Paternal)	rs750213588	0.00002094	*DNAH10*	3
NC_000017.11:g.10459346C>T	NP_060003.2: p.(Val498M)	missense (25.7)	**22.3-22.4** (Paternal)	rs143641715	0.00009777	*MYH4*	3
NC_000017.11:g.39175200A>T	NP_000714.3: p.(Ile597Asn)	missense (23.8)	**22.3-22.4** (Paternal)	.	.	*CACNB1*	3
NC_000017.11:g.63945452C>T	NP_000325.4: p.(Glu1210Lys)	missense (19.44)	**22.3-22.4** (Paternal)	rs761135349	0.00002094	*SCN4A*	3
NC_000020.11:g.53953667G>A	NP_003648.2: p.(Thr482Ile)	missense (17.26)	**22.3-22.4**-22.5 (Maternal)	.	.	*BCAS1*	3
NC_000003.12:g.175097095A>G	NP_996898.2: p.(Ser117Gly)	missense (18.11)	**22.3-22.4** (Paternal)	rs746178036	.	*NAALADL2*	3
NC_000006.12:g.166459476G>T	NP_066958.2: p.(Pro350Thr)	missense (18.58)	**22.3-22.4**-22.5 (Paternal)	.	.	*RPS6KA2*	3
NC_000007.14:g.128030912C>T	.	splicing (.)	**22.3-22.4**-22.5 (Paternal)	.	0.000006982	*LRRC4*	3
NC_000008.11:g.143867209insC	NP_112598.3: p.(Val2016Cysfs*82)	frameshift insertion (.)	**22.3-22.4** (Maternal)	rs781861594	0.00002817	*EPPK1*	3
NC_000006.12:g.152453654C>T	NP_892006.3: p.(Asp987Asn)	missense (24.4)	**22.3-22.4**-22.5 (Paternal)	.	0.00001396	*SYNE1*	3-S
NC_000013.11:g.100209381C>G	NP_000273.2: p.(Ala173Gly)	missense (23)	**22.3-22.4** (Paternal)	rs750928504	0.00002094	*PCCA*	S
NC_000015.10:g.28142369G>C	NP_004658.3: p.(Leu3857Val)	missense (23)	**22.3-22.4**-22.5 (Paternal)	.	.	*HERC2*	S
**b) Family 105**							
NC_000007.14:g.142771008T>C	*NP_001290343.1: p.(Phe9Ser)*	*missense (19.74)*	***105.3*** *(De novo)*	*rs757333739*	*0.00008845*	*PRSS2*	.
NC_000011.10:g.76544332T>C	NP_001287871.1: p.(Met943Thr)	missense (24.9)	**105.3** (Paternal)	.	.	*EMSY*	2
NC_000014.9:g.93621924A>T	NP_001333147.1: p.(Asn1614Ile)	missense (22.5)	**105.3** (Maternal)	.	.	*UNC79*	2
NC_000016.10:g.1205207C>T	NP_066921.2: p.(Pro849Ser)	missense (18.22)	**105.3** (Paternal)	rs370675810	0.0000977	*CACNA1H*	2
NC_000005.10:g.66054545G>T	NP_001240626.1: p.(Arg1076Leu)	missense (27.5)	**105.3** (Paternal)	rs774967294	0.000006983	*ERBIN*	2
NC_000001.11:g.34868751C>T	NP_001073887.1: p.(Arg780His)	missense (22.6)	**105.3** (Maternal)	rs745644846	0.000006979	*DLGAP3*	3
NC_000019.10:g.1045174G>C	NP_061985.2: p.(Arg463Pro)	missense (22.9)	**105.3** (Paternal)	rs3752233	0.0002	*ABCA7*	3
NC_000002.12:g.169259049G>A	NP_004516.2: p.(Ser830Leu)	missense (23)	**105.3** (Maternal)	rs767925581	0.000014	*LRP2*	3
NC_000002.12:g.	NP_997198.2:	missense	**105.3**	.	.	*CMPK2*	3

6865123C>T	p.(Val192Ile)	(16.75)	(Maternal)				
NC_000021.9:g.3 3797479G>A	NP_003015.2: p.(Glu685Lys)	missense (21.7)	**105.3** (Maternal)	rs769546913	.	*ITSN1*	3

Supplementary Table 3. Summary of clinical data of ASD individuals carrying *CACNA1H* biallelic variants.

	**Proband 22.3**	**Proband 22.4**	**Proband 105.3**
**Age at first assessment**	40 months	40 months	40 months
**Sex**	F	F	M
**Morphology**			
Growth at birth<br>*Head circumference (HC)*	weight =2.021 kg<br>HC: 50 *%ile*	weight =1.984 kg<br>HC: 50 *%ile*	weight = 3.5 Kg<br>HC: 75 *%ile*
**Neurodevelopment**			
Non-verbal brief IQ (Leiter-R)	36 (severe intellectual disability)	48 (moderate intellectual disability)	48 (moderate intellectual disability)
Speech development	Lack of speech	Lack of speech	Delayed and atypical speech
**Autism scales**			
CARS2-ST total score (ASD diagnosis cut-off = 30)	48.5	43	39
ADOS-2 (module 1) autism features level	High (8)	High (9)	High (8)
**DSM-5 severity level**	3 ("requiring very substantial support")	3 ("requiring very substantial support")	3 ("requiring very substantial support")
**Neurological**			
Epilepsy	Four febrile convulsions and two apparently generalized convulsive seizures without fever	-	-
EEG	Focal (right mid-posterior) and diffuse paroxysmal abnormalities	Normal	Frequent multifocal spike-waves in the left central and in the right centro-temporal regions, increased in the early stages of sleep
Brain MRI (1.5 Tesla)	Normal	Normal	Normal
